# Structural Analysis of Intermolecular Interactions in the Kinesin Adaptor Complex Fasciculation and Elongation Protein Zeta 1/ Short Coiled-Coil Protein (FEZ1/SCOCO)

**DOI:** 10.1371/journal.pone.0076602

**Published:** 2013-10-07

**Authors:** Marcos Rodrigo Alborghetti, Ariane da Silva Furlan, Júlio César da Silva, Maurício Luís Sforça, Rodrigo Vargas Honorato, Daniela Campos Granato, Deivid Lucas dos Santos Migueleti, Jorge L. Neves, Paulo Sergio Lopes de Oliveira, Adriana Franco Paes-Leme, Ana Carolina de Mattos Zeri, Iris Concepcion Linares de Torriani, Jörg Kobarg

**Affiliations:** 1 Laboratório Nacional de Biociências-LNBio, Centro Nacional de Pesquisa em Energia e Materiais-CNPEM, Campinas, SP, Brasil; 2 Departamento de Bioquímica-Programa de Pós-graduação em Biologia Funcional e Molecular, Instituto de Biologia, Universidade Estadual de Campinas, Campinas, SP, Brasil; 3 Departamento de Genética, Evolução e Bioagentes, Programa de Pós-graduação em Genética e Biologia Molecular, Instituto de Biologia, Universidade Estadual de Campinas, Campinas, SP, Brasil; 4 Condensed Matter Physics Department, Instituto de Física “Gleb Wataghin”, Universidade Estadual de Campinas, UNICAMP, Campinas, SP, Brasil; University of Oulu, Finland

## Abstract

Cytoskeleton and protein trafficking processes, including vesicle transport to synapses, are key processes in neuronal differentiation and axon outgrowth. The human protein FEZ1 (fasciculation and elongation protein zeta 1 / UNC-76, in *C. elegans*), SCOCO (short coiled-coil protein / UNC-69) and kinesins (e.g. kinesin heavy chain / UNC116) are involved in these processes. Exploiting the feature of FEZ1 protein as a bivalent adapter of transport mediated by kinesins and FEZ1 protein interaction with SCOCO (proteins involved in the same path of axonal growth), we investigated the structural aspects of intermolecular interactions involved in this complex formation by NMR (Nuclear Magnetic Resonance), cross-linking coupled with mass spectrometry (MS), SAXS (Small Angle X-ray Scattering) and molecular modelling. The topology of homodimerization was accessed through NMR (Nuclear Magnetic Resonance) studies of the region involved in this process, corresponding to FEZ1 (92-194). Through studies involving the protein in its monomeric configuration (reduced) and dimeric state, we propose that homodimerization occurs with FEZ1 chains oriented in an anti-parallel topology. We demonstrate that the interaction interface of FEZ1 and SCOCO defined by MS and computational modelling is in accordance with that previously demonstrated for UNC-76 and UNC-69. SAXS and literature data support a heterotetrameric complex model. These data provide details about the interaction interfaces probably involved in the transport machinery assembly and open perspectives to understand and interfere in this assembly and its involvement in neuronal differentiation and axon outgrowth.

## Introduction

Neuronal differentiation *in vitro* and *in vivo* involves coordinated changes in various levels, including gene expression, the cellular cytoskeleton and protein trafficking processes [1]. During the neuronal differentiation, the growth of neurite processes from the cell body involves a massive increase in cell surface area [2]. The membrane increment occurs in a very dynamic axon structure called growth cone. For growth cones to extend, vesicles derived from the Golgi apparatus fuse with the plasma membrane by a process of regulated exocytosis [3]. These vesicles are not only transported but are also differentially sorted into dendrites or axons [4,5]. Localization of dendritic, axonal and pre-synaptic proteins is dependent of kinesin mediated transport [6]. Knockdown of the kinesin motors using antisense oligonucleotides not only disrupts dendritic or axonal protein localization of these proteins, but also suppresses neurite outgrowth, presumably by blocking kinesin-dependent vesicle transport [7,8].

Classical mutant screens in the model organism *Caenorhabditis elegans* led to the discovery of genes involved in these processes. Since some of these mutations cause defects in locomotion their respective genes were termed as *unc* (for uncoordinated). Several *unc* mutations affect molecular motor dependent intracellular transport and thereby vesicle cargo delivery, including some kinesins as UNC104/KIF1A, UNC-116/kinesin heavy chain (KHC) and kinesin light chain KLC-2 that are core proteins of the motor transport. UNC-76 and UNC-69 directly interact with each other and were identified as crucial components of this multi-protein machinery [9,10], since UNC-76 also interacts directly with kinesin1 [11]. So, a general picture is emerging that kinesin family members link to their cargoes via adaptor/scaffolding proteins [12], such as UNC-76/-69. Recent studies with the human proteins FEZ1 (=UNC-76) and SCOCO (=UNC-69) further support this notion, since FEZ1 was described as a hub protein, which interacts through its C-terminal coiled-coil region with over 80 different proteins, of different functional classes, that may represent cargoes [11,13,14]. Furthermore, FEZ1 knockdown causes mitochondria mislocalization [15] and vesicle accumulation in the soma of PC12 cells [16], which could be explained by a failure in the transport of these organelles. Furthermore, FEZ1 is the first protein identified that binds to the inhibitory globular tail of KHC, and together with JIP1 (that binds to KLC), release the kinesin-1 from the auto-inhibitory state. These data indicate that FEZ1 is not only a cargo or an adaptor, but also acts in Kinesin-1 activation [12].

Previous work from our group demonstrated that FEZ1 has regions with characteristics of natively unfolded protein [17], and forms disulfide-bridge stabilized homo-dimers in solution [18]. The data suggest that FEZ1 dimerizes through its N-terminal region but exposes its C-terminal regions, which contain the coiled-coil interaction modules. This present work focuses on the interaction interfaces involved on FEZ1 homodimerization through its N-terminal region and on interaction interfaces involved in complex formation with the canonical protein interaction partner SCOCO (short coiled-coil protein) [10] through its C-terminal region. Furthermore, we proposed a global and generic model of this complex. The conjunction of these data led us to hypothesize that FEZ1 acts as a dimeric and bivalent transport adaptor that connects the kinesins to a large and diverse group of cargo molecules, complexes or even organelles.

## Experimental Procedures

### Cloning and protein purification

The cloning of FEZ1 (92-194) used in NMR studies is described in Alborghetti et al. (2010) [18]. pET-FEZ1 (92-194) plasmid was transformed into BL21(DE3) *Escherichia coli* which weregrown at 37°C in M9 minimal medium supplemented with 30 µg/mL kanamycin, 4 g/L [13C]glucose and 1 g/L [15N]ammonium chloride. Expression was induced at an OD_600_ of 0.8-1.0 by the addition of 0.2 mM IPTG for 24 h. The cells were harvested by centrifugation at 5000 g for 10 min and the purification methodology was previously described in Alborghetti et al. (2010) [18]. Fractions containing pure 6xHis-FEZ1 (92-194) were pooled and concentrated to 0.3 mM in buffer containing 20 mM phosphate, 50mM NaCl, pH 6.2. The sample was divided in two aliquots, one aliquot was reduced with 4 mM DTT, and both were analyzed by NMR spectroscopy.

The nucleotide sequence of SCOCO (2-82) (accession EAX05104) in the vector pACT2 – retrieved from a yeast two-hybrid system assay with FEZ2 (207-353) as bait [14] – was sub-cloned into the bacterial expression vector pGEX-4T-2 (GE Healthcare, Waukesha, WI) between restriction sites *Eco*RI and *Xho*I to allow expression of recombinant GST fusion proteins and sub-cloned into pET28a to allow expression of recombinat 6His proteins in *Escherichia coli* BL21 (DE3) cells. The cells were induced at OD 0.7 to express protein for 4 h at 37°C using 0.5 mM isopropyl 1-thio-β-D-galactopyranoside. For cross-linking studies, the protein 6His-FEZ1 (1-392) (accession AAC51282) expression and purification was performed as described by Assmann et al. [13]. The 6His-SCOCO (2-82) protein purification followed the same purification protocol for 6His-FEZ1 (1-392) [17].

For SAXS experiments, cells from 2L of bacterial culture for 6His-FEZ1 (1-392) and 1L for GST-SCOCO (2-82) were harvested together by centrifugation at 4,500*g* for 10 min, and the cell pellet was ressuspended and incubated for 30 min with 10 volumes of lysis buffer (137 mM NaCl, 2.7 mM KCl, 10 mM Na_2_HPO_4_, 1.8 mM KH_2_PO_4_, pH 7.4, 1 mg/ml lysozyme, 1 mM phenylmethylsulfonyl fluoride, and 0.05 mg/ml DNase). After three cycles of sonication, soluble and insoluble fractions were separated by centrifugation at 28,500*g* for 30 min at 4°C. The cleared supernatant was then loaded onto a HiTrap chelating column (GE Healthcare) pre-equilibrated with lysis buffer (lacking lysozyme and DNase), followed by extensive wash of the column with the same buffer. Bound proteins were eluted in a gradient of 0–100% of elution buffer (137 mM NaCl, 2.7 mMKCl, 10 mM Na_2_HPO_4_, 1.8 mM KH_2_PO_4_, 1 mM phenylmethylsulfonyl fluoride, and 500 mM imidazole, pH 7.4). Aliquots of each eluted fraction were analyzed by SDS-PAGE, and peak fractions containing 6His-FEZ1 (1-392) and GST-SCOCO (2-82) were submitted to a new step of purification in a GST-Trap column (GE Healthcare). Again, aliquots of each eluted fraction obtained were analyzed by SDS-PAGE and 1 mL aliquot was dialyzed with buffer (137 mM NaCl, 2.7 mMKCl, 10 mM Na_2_HPO_4_, 1.8 mM KH_2_PO_4_) and analyzed by DLS (Dynamic Light Scattering). The aliquots that showed a percentage of poly-dispersity indicative of a monodisperse solution were selected for SAXS data acquisition. The concentration of the recombinant protein was spectroscopically determined using the calculated extinction coefficient for the denatured proteins [19].

### NMR Spectroscopy

NMR experiments for sequential assignment were performed at 293 K using a Agilent/Varian Inova 600 MHz spectrometer equipped with a cryogenic probe. The following experiments were recorded: 15N-HSQC; 15N-edited NOESY-HSQC 150 ms mixing time; 15N-edited TOCSY-HSQC; HNCA; HN(CO)CA; HNCACB; CBCA(CO)NH; HNCO; HN(CA)CO [20]. All spectra were processed using NMRPipe [21] and analysed by NMRview [22] and Smartnotebook software [23].

### Relaxation Measurements

For the backbone amide relaxation measurements, 15N T1, 15N T2 and heteronuclear NOE experiments were recorded at a 15N frequency of 60.78 MHz on a Varian Inova 600 MHz spectrometer equipped with a cryogenic probe. T1 relaxation delays were set to 10, 210, 410, 810, 1010, 1410, 1610, 1810, 2010, and 2410 ms. T2 relaxation delays were set to 10, 30, 50, 70, 90, 110, 150, 170, 210 and 250 ms. In all the experiments a relaxation delay of 3 s was used. For the heteronuclear NOE measurements, a pair of spectra was recorded with and without proton saturation. Spectra recorded with proton saturation utilized a 3 s recycle delay followed by a 5 s period of saturation, while spectra recorded in the absence of saturation employed a recycle delay of 8 s. Peak volumes were fitted to a single exponential decay function using the program NMRView. The two-dimensional experiments were acquired with 2048 x 256 complex points [20].

### Cross-linking Analysis between 6xHis-FEZ1 (1-392) and 6xHis-SCOCO (2-82) by Mass Spectrometry

Cross-linking reactions were performed as described in Aragão et al. (2012) [24]. In summary, 5 x 10^-10^ mol of each purified recombinant protein was incubated with 1.25 mM disuccinimidyl suberate (DSS, spacer arm length: 11.4 Å, Sigma-Aldrich) for 2 h at room temperature, followed by quenching with Laemmli sample buffer. DSS-cross-linked complexes were identified as shifted bands in 12% SDS-PAGE. The protein complexes of each band were digested with trypsin. The samples were dried in a vacuum concentrator and reconstituted in 100 µl of 0.1% formic acid. 4.5 µl of the resulting peptide mixture was analyzed in LTQ Velos Orbitrap. For protein identification, peak lists (msf) were generated from the raw data files using Proteome Discoverer version 1.3 (Thermo, Fisher Scientific) with Sequest search engine and searched against Human International Protein Database, version 3.86 (91,522 sequences; 36,630,302 residues, release July 2011) with carbamidomethylation (+57.021 Da) as fixed modification, oxidation of methionine (+15.995 Da), and chemical cross-linked with DSS (mass of dead-end cross-linking, 156.07864 Da) as variable modifications, one trypsin missed cleavage, and a tolerance of 10 ppm for precursor and 0.02 Da for fragment ions.

For cross-linked analysis, the raw data files generated by Xcalibur version 2.1 (Thermo, Fisher Scientific) were converted to a peak list format (mgf) using Proteome Discoverer version 1.3 (Thermo, Fisher Scientific). The mgf files were analyzed in MassMatrix software [25] to automatically search chemical cross-linkage against databases containing the FEZ1 and SCOCO amino acid sequences, according to the software instructions. The parameters for cross-linking analysis used in MassMatrix software were carbamidomethylation (+57.021 Da) as fixed modification, oxidation of methionine (+15.995 Da) as variable modifications, chemical cross-linked with DSS (138.06808 Da) noncleavable by enzymes, four trypsin missed cleavages, and a tolerance of 10 ppm for precursor and 0.02 Da for fragment ions. Search results with high confidence (Mass-Matrix pp score >30) and potential cross-linked peptides were manually validated for b and y ion series containing α and β chains [25,26]. This experiment was performed two times.

### In silico modelling

Protein modeling algorithms were applied to obtain three dimensional models of both FEZ1 and SCOCO. First, we applied a homology modeling protocol using YASARA (Z-score = -2.214) [27] to build the FEZ1 structure based on the templates obtained from Blast search against a sequence database extracted from Protein Databank (PDB) [28]. Structure of SCOCO was obtained using an ab initio approach of I-TASSER [29] with C-score of -0.93. The interaction complex between FEZ1 and SCOCO was built using a previously developed docking method using geometrical minimization routines plus crosslinking experimental data [24]. The dimer was predicted using the above docking algorithm with NMR data as restraints.

### SAXS experiments, data reduction and calculation of molecular parameters

The SAXS experiments were performed at the D02A-SAXS2 beamline of the Laboratório Nacional de Luz Síncrotron (LNLS, Campinas, Brazil). Measurements were performed with a wavelength of λ = 1.488 Å. and a 165-MARCCD area detector. Two sample-to-detector distances were used (902.1 and 1504.9 mm), that provided data corresponding to a momentum transfer range of 0.08 < q < 0.30 Å^-1^ where q is the magnitude of the q-vector defined by q = (4π/λ)sinθ (2θ is the scattering angle). For X-ray exposures the protein samples were placed in a temperature-controlled (T = 20°C) 1-mm path length cell with mica windows [30]. Three successive frames of 300 s each were recorded for each sample. The buffer scattering data were recorded for 300 s before and after each sample data collection and averaged thereafter. The scattering curves were individually corrected for detector response and scaled by incident beam intensity and sample absorption. The averaged buffer signal was subtracted from the corresponding sample scattering. The intensity curves were carefully checked for possible radiation-induced damage to the sample. The scattering patterns of the complex were recorded from two solutions with concentrations 1.10 mg/mL and 0.34 mg/mL. The molecular masses of all samples were estimated from the extrapolated values of their SAXS intensity at the origin I(0) and the corresponding I(0) value of bovine serum albumin (BSA) in solution used as a reference [31,32]. The radius of gyration of the complex was first roughly estimated using the Guinier approximation [33]. When dealing with unstructured proteins, for which the Guinier approximation holds true on a very restricted validity range, (corresponding to q < 1/Rg), Debye’s equation is generally applied because it adequately describes the scattering in the domain (q < 1.4/R_g_) [34,35]. The radius of gyration can also be evaluated from the pair distance distribution function p(r). In this work, the p(r) function was calculated by the indirect transform package GNOM [36] used for the entire curve fitting procedure. The p(r) function provided an approximate value of the maximum dimension D_max_ of the molecules (where its value reached zero) and was used by the program for the calculation of Rg.

## Results

### Unambiguous NMR signals indicate that FEZ1 homodimerizes in an anti-parallel fashion

**Figure 1:**
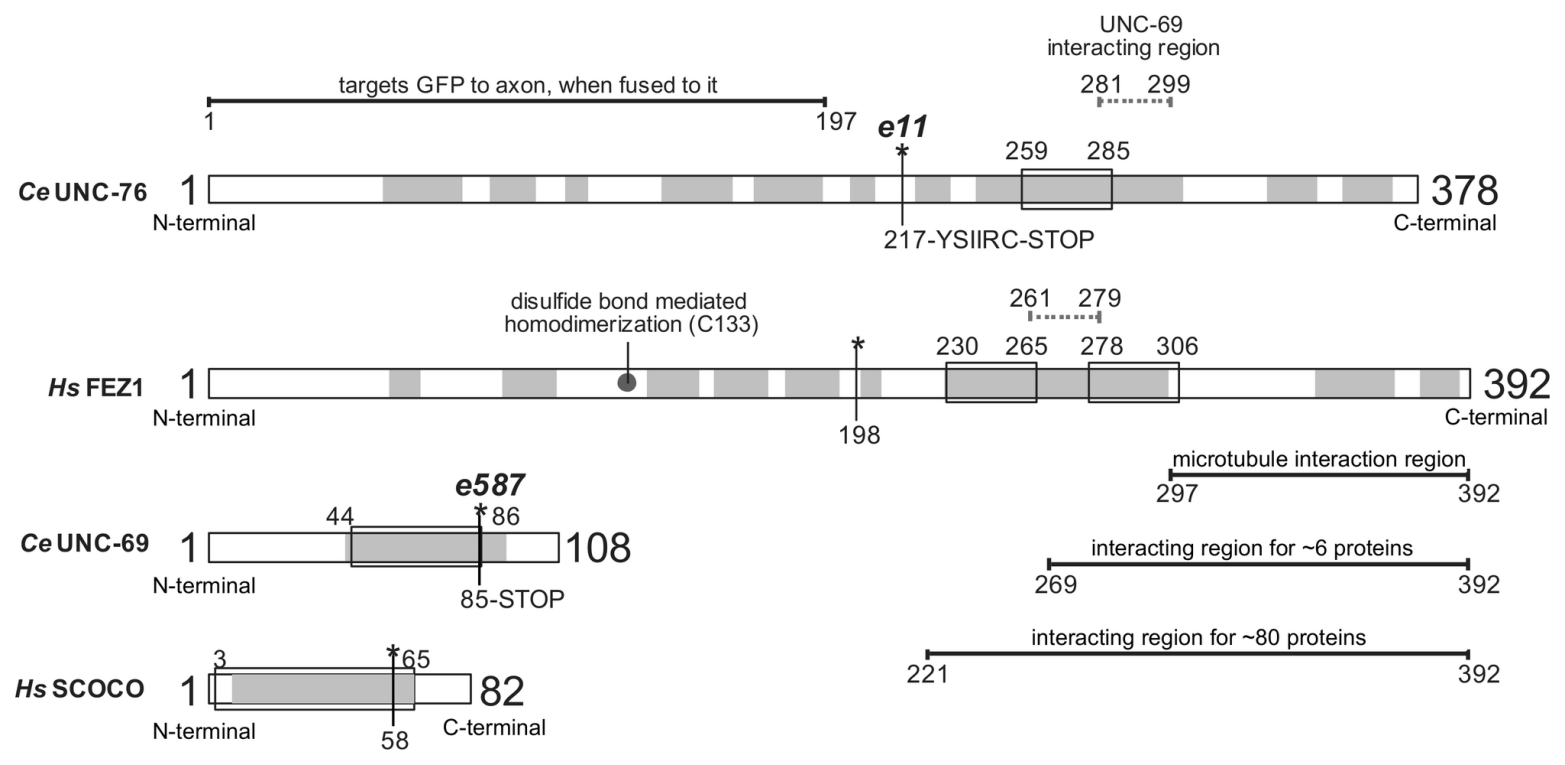
General scheme of UNC-76/FEZ1 (*C. elegans / H. sapiens*) and UNC-69/SCOCO (*C. elegans / H. sapiens*) proteins. Mutations observed in *C. elegans*, and the corresponding region in the human protein, are indicated by asterisks. Regions of interaction with other proteins are indicated. Coiled-coils: box, alpha-helix prediction: gray.

**Figure 2:**
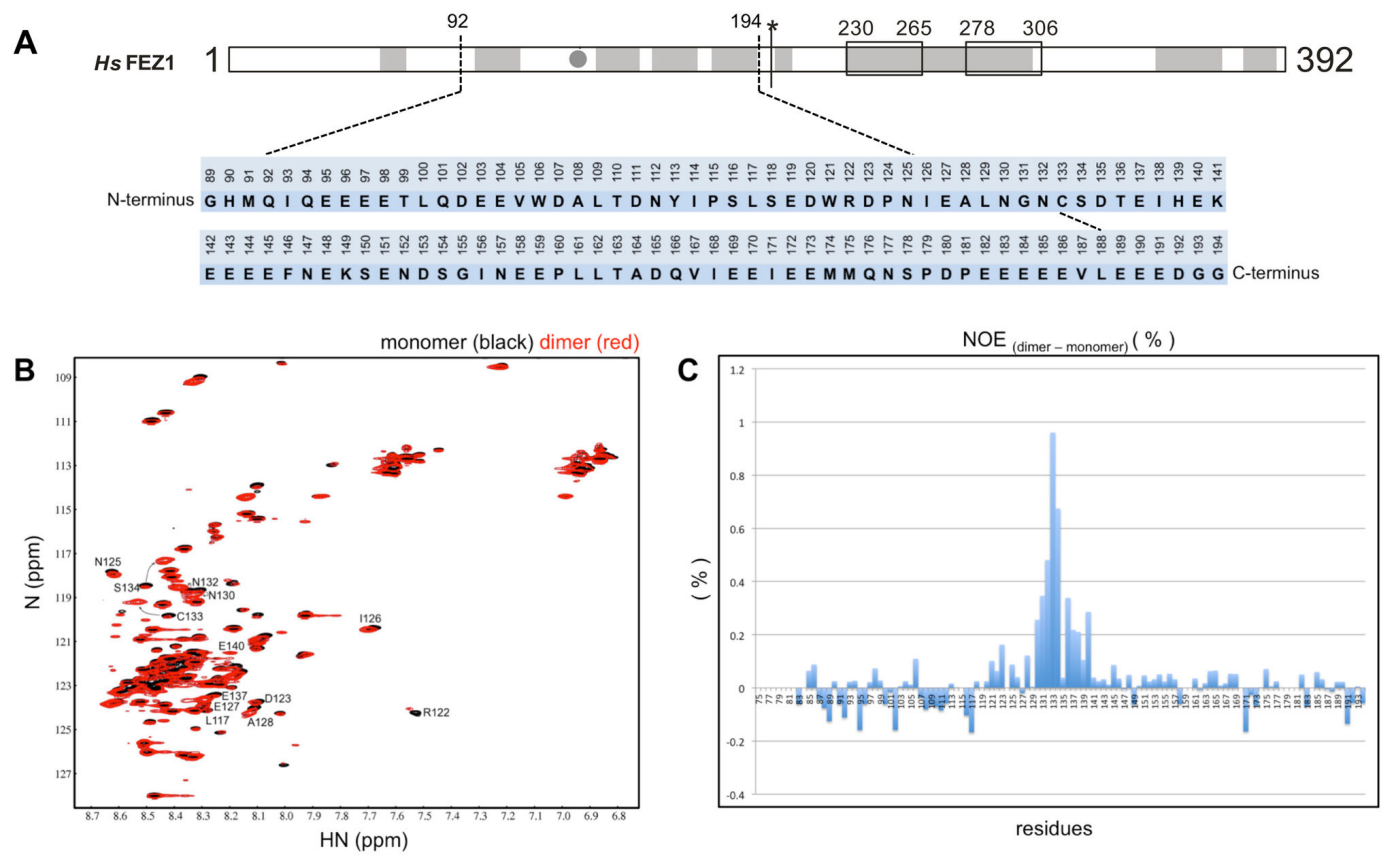
FEZ1 homodimerization involves few amino acids residues. A) Amino acid sequence of human FEZ1 protein (92-194). The numbers indicate the position of each amino acid residue within the full-length sequence. The first three residues, unnumbered, are generated by the recombinant protein’s cleavage with TEV protease. B) 15N-HSQC FEZ1 (92-194) Nuclear Magnetic Resonance (NMR) spectra. HSQC shows chemical shifts in reduced monomeric protein (black) and non-reduced dimeric protein (red). The spectrum was obtained in spectrometer 600 MHz. For the series of experiments, isotope 15N was introduced in minimal medium for growth of bacteria and induction of protein expression. C) steady-state heteronuclear NOE experiments with dimers and monomers. Heteronuclear NOEs intensities of the monomer were subtracted from those of the dimer, resulting in the differential pattern of relaxation corresponding to amino acids probably present in the region of homodimerization.

**Figure 3:**
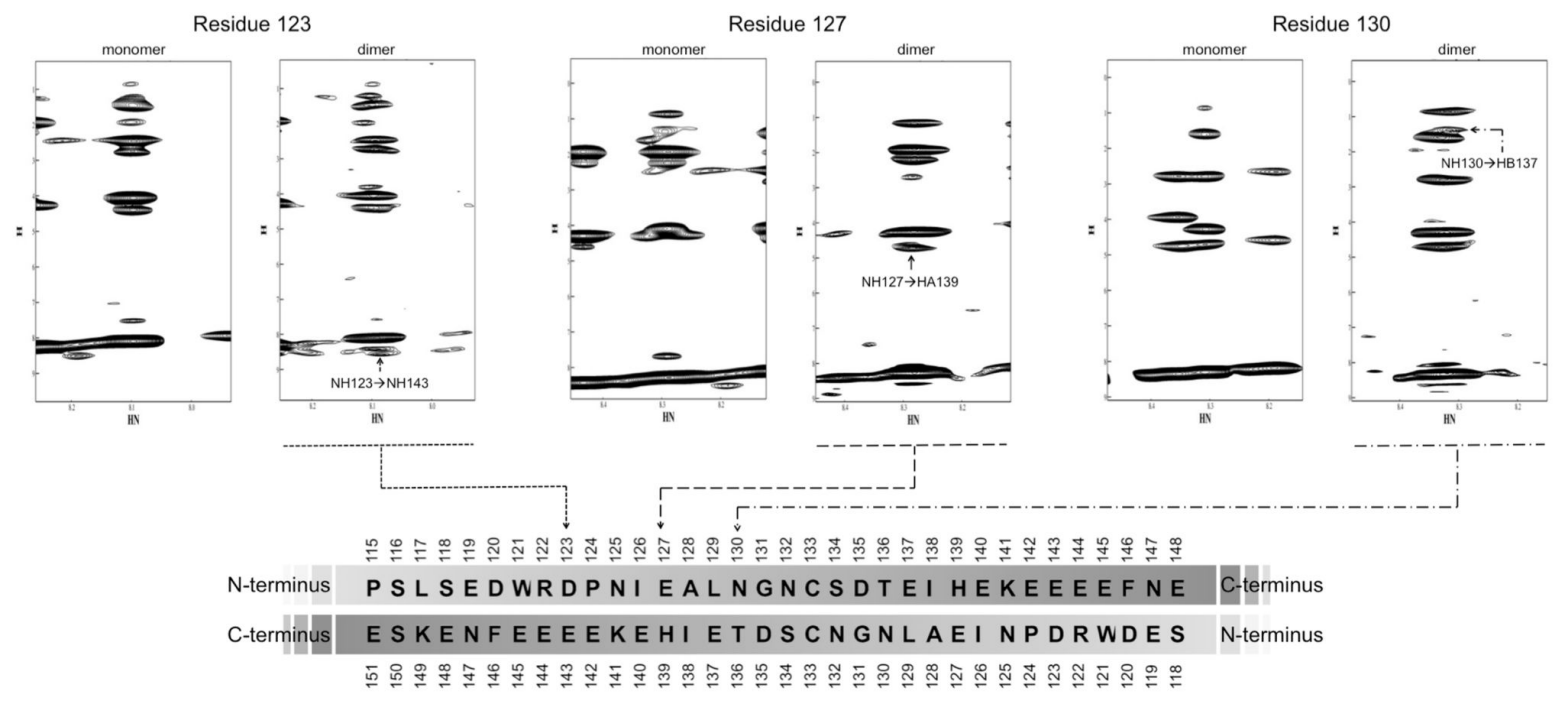
FEZ1 is an anti-parallel dimer. 15N-edited NOESY-HSQC of dimers and monomers of FEZ1 (92-194) shows contacts between side chains of amino acids that occur in the homodimer, preferably in an anti-parallel topology. These contacts occur between residues 123 and 143, 127 and 139, 130 and 137, and are indicated in the sequence shown at the bottom of the figure.

In 2010, Alborghetti and collaborators showed that human FEZ1 protein forms a disulfide bond mediated dimer in its N-terminal region [18] (Figure 1). We accessed amino acid contacts involved in this interaction by NMR. The amide resonances of the amino acids in well resolved regions of the spectrum were assigned using the monomeric protein FEZ1 (92-194) (Figure 2A) that had been double-labeled with N15 and C13. The 2D 15N-HSQC spectra of the protein FEZ1 (92-194) in its monomeric conformation (with 4mM DTT, in black) and dimeric conformation (without DTT, in red) are superimposed in Figure 2B. Although both proteins are for the most part disordered, as is indicated by the clustering of peaks in the 8.4 ppm region of the spectrum, we were able to detect some distinct chemical shifts, that allowed us to identify several residues involved in inter-chain contacts (Figure 2B). In addition to the 15N-HSQC data a relaxation data analysis allowed the definition of a relative defined region in the amino acid sequence that is involved in the dimerization (Figure 2C). Heteronuclear NOEs are sensitive to movements in the picosecond to nanosecond timescale and reduced signal intensities are indicative of absence of restrictive dynamics in this time range. Subtraction of the dimer heteronuclear NOEs intensities signals from those of the monomer resulted in the identification of a region with more restricted dynamics that clusters around the region of Cys133 and stretches from amino acid residues 130 to 140 (Figure 2C). Comparison of NOESY spectra from dimers and monomers resulted in the identification of three unambiguous signals derived from direct inter-chain contacts that involve pairwise contacts of D123 and E143, E127 and H139, and N130 and E137, suggesting that the homo-dimerization occurs in an anti-parallel fashion (Figure 3). Contacts between the backbone of amino acids with the same charge, as in the case of D123 and E143, are observed. The approximation of these residues may occur through the interaction of neighboring residues that although assigned, were not included as unambiguous peaks suggesting an antiparallel dimerization, as for E143 and D123. Most interestingly, these findings confirm our previous finding [18] that Cys133 of both monomers form a disulfide bond, since in Figure 3 we can see that both Cys are in phase with the anti-parallel arrangement generated by the three pairwise contacts mentioned above.

### Human FEZ1 and SCOCO interaction is similar to that of the UNC-76 and UNC-69 complex from *C. elegans*

**Figure 4:**
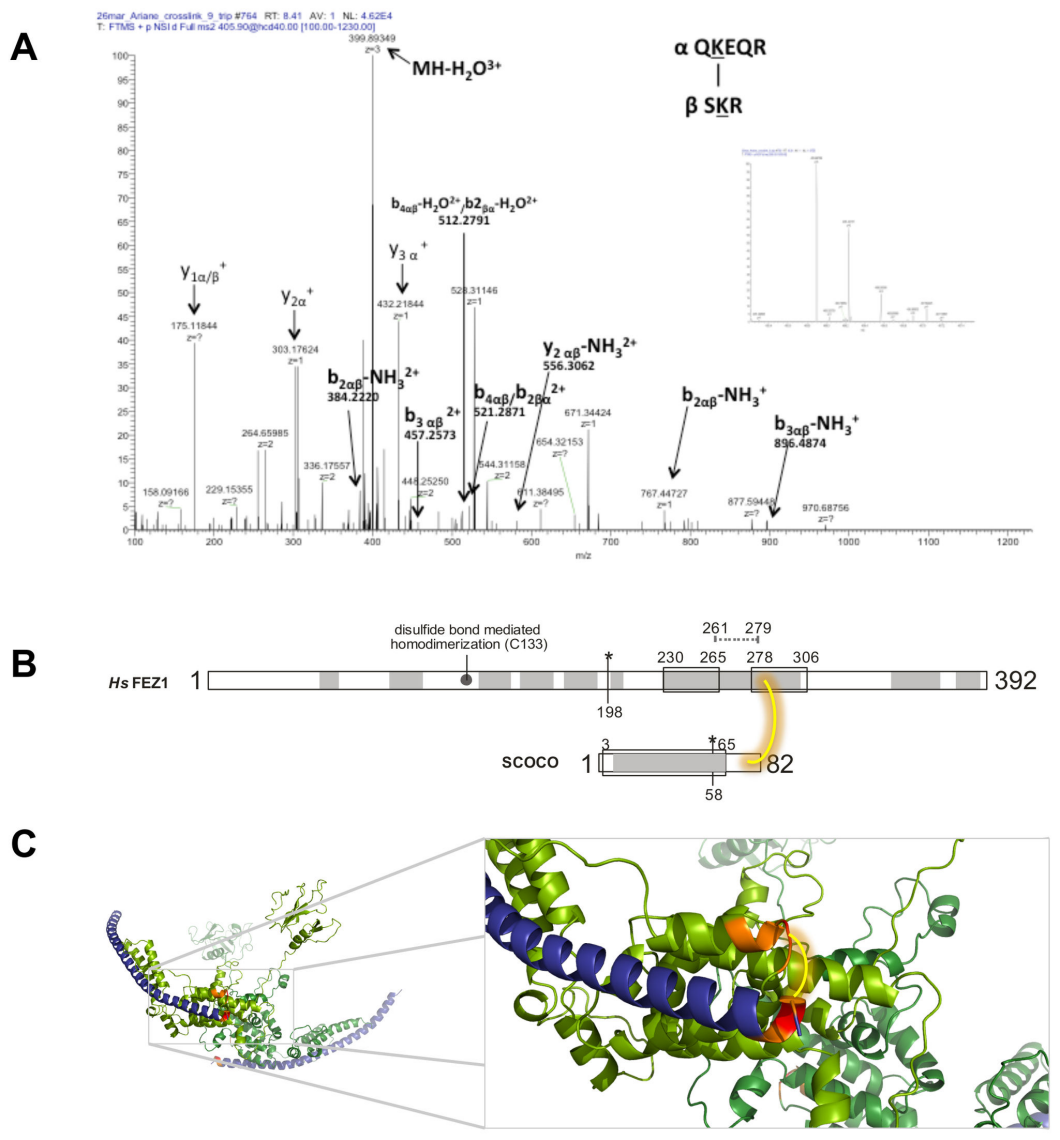
Interaction between FEZ1 and SCOCO. A) Purified recombinant proteins FEZ1 and SCOCO were incubated, chemically cross-linked, digested with trypsin, and analyzed by MS. MS/MS spectra were manually validated for b and y ion series of the α (peptide of FEZ1) and β (peptide of SCOCO) chains. B) General scheme of FEZ1 and SCOCO proteins cross-linked. Coiled-coils: box, alpha-helix prediction: gray. Amino acids 261-279 in FEZ1 correspond to the mininal interaction region of UNC-69/SCOCO in UNC-76/FEZ1.C) Best conformation based on both cross-link distance and energy value of the in silico modeled complex. FEZ1 is colored in green, and SCOCO is depicted in deep blue. The peptides identified in the MS analysis are shown in orange and the lysine residue in red. DSS is represented in yellow.

Su and collaborators (2006) showed that UNC-76 protein (orthologue of FEZ1 in *C. elegans*) interacts with UNC-69 protein (orthologue of SCOCO in *C. elegans*) via coiled-coils interactions, and they determined the minimal UNC-69-interaction region in UNC-76 (amino acids 281-299) [10] (Figure 1). However, the minimal interaction region in UNC-69 was not determined. Exploring the human orthologues, we expressed and purified the recombinant proteins 6xHis-FEZ1 (1-392) and 6xHis-SCOCO (2-82). To evaluate the interaction interface between 6xHis-FEZ1 and 6xHis-SCOCO, we chemically cross-linked the recombinant purified proteins. Initially, the cross-linked proteins were separated by SDS-PAGE, and the three main bands of >170, ~130, and ~70 KDa were digested with trypsin. After manually verifying possible hits from MassMatrix search results, we identified one cross-link with high confidence (MassMatrix pp score >30). The ~130KDa band showed an identification for FEZ1 and SCOCO by the tryptic peptides^280^QKEQR^284^ and^79^SKR^81^, respectively, cross-linked by the side chains of the underlined lysine residues. The identified FEZ1 peptide is adjacent to the minimal interaction region determined in UNC-76. This experiment was repeated two times, and the same cross-linked peptides were confirmed. The manual validation of cross-linked peptides is shown in the Figure 4A and Figure S1 in File S1. The lysine cross-linked peptides identified by mass spectrometry are highlighted in the predicted region of interaction of FEZ1 and SCOCO in Figure 4B and in the in silico modeled complex shoen in Figure 4C (see details below).

The fact that a mutation of *unc69* in *C. elegans*, involving a premature stop codon at position Q86, causes problems in vesicle transport, neuritogenesis and consequently the paralyzed phenotype of the worm [10] (Figure 1) lead us to hypothesize that this C-terminal region (a coil) could be interacting with UNC-76/FEZ1. We generated an equivalent mutation of human SCOCO where the last 18 amino acids have been deleted in order to avoid disrupting the region predicted to coiled-coil, and tested for interaction with FEZ1 in the yeast-two hybrid system. Surprisingly, both proteins still interact (Figure S2 in File S1), suggesting that human SCOCO with mimetic mutation present in *unc69* mutant worm still interacts with FEZ1 protein. Interestingly, the corresponding minimal interaction region identified in UNC-76 present in FEZ1 in the model (Figure S2 in File S1) is adjacent to both SCOCO region 2-82 and the deleted fragment 65-82, suggesting that the mutant phenotype is not dependent on the loss of interaction between FEZ1 and SCOCO, but possibly rather on other mechanisms maybe related to the interaction of additional components with SCOCO.

### FEZ1 homodimer interacts with two SCOCO molecules

**Figure 5:**
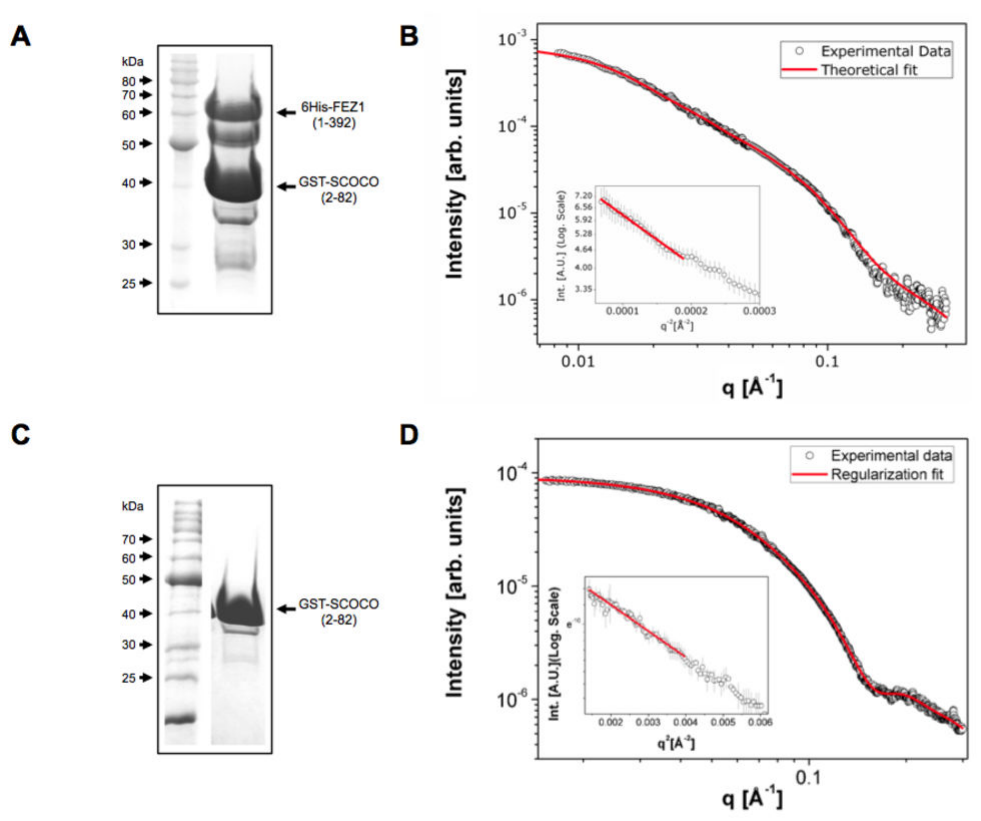
Purification of the FEZ1-SCOCO complex and GST-SCOCO and SAXS experimental data A) SDS-PAGE 10% of the 6His-FEZ1 (1-392) and GST-SCOCO (2-82) protein complex. The complex was analyzed by SAXS at 1.10 mg/mL in PBS buffer solution. The complex poly-dispersity was 28,0% according to DLS assay. B) SAXS (Small Angle X-ray Scattering) experiments of 6His-FEZ1 (1-392) interacting with GST-SCOCO (2-82). C) SDS-PAGE 10% of the GST-SCOCO (2-82) protein. D) The protein was analyzed by SAXS at 0.84 mg/mL in PBS buffer solution. The figure shows the experimental intensity points (empty symbols) and the theoretical fit (continuous line) obtained with the GNOM program package for both samples. Insets in both panels (B) and (C) show the linear behavior of the data in the Guinier region. R_g_ and D_max_ values obtained from the GNOM fitting for GST-SCOCO and FEZ1-SCOCO samples were (28.7 ±1, approx. 95 Ǻ) and (107±1, approx. 340 Ǻ), respectively. SAXS data of FEZ1 protein were previously published [17].

For SAXS studies, we reconstituted the FEZ1-SCOCO complex expressing 6xHis-FEZ1(1-392) and GST-SCOCO (2-82) in *E. coli* (DE3) separately, performing the lysis of both pellets together and *in vitro* complex-formation followed by a two-step co-affinity purification, exploring first the 6×His tag and afterwards the GST fusion (Figure 5A). Next steps involved SAXS data acquisition and analyses (Figure 5B). We opted to fuse the small protein SCOCO (9 KDa) with GST (26 KDa) in order to facilitate its detection within the complex. From the SAXS data we calculated that the observed complex had an approximate molecular weight of 172 KDa, which is in good agreement with the formation of a hetero-tetrameric complex composed of two molecules of 6xHis-FEZ1 interacting with two molecules of GST-SCOCO. This value is very close to the predicted molecular weight of 171.2 KDa, which is the sum of 2 x 48.8 KDa (6xHis-FEZ1) and 2 x 36.8 KDa (GST-SCOCO). However, we must consider the dimerization of GST in this analysis [37-39]. To access exclusively the oligomeric state of the GST-SCOCO molecule when interacting with FEZ1 protein is not trivial because FEZ1 protein has many regions intrinsically disordered and most methods that allow us to estimate the molecular mass use globular proteins as standards. The estimated masses determined by our SAXS measurements together with the detection of the complex FEZ1/SCOCO in a 2:2 stoichiometric ratio by McKnight [40] suggest that this complex exists in a heterotetrameric state. Furthermore, GST-SCOCO SAXS measurements shows a protein in monomeric configuration (Figure 5C and Figure 5D) with a molecular weight of 37.43 KDa.

We performed in silico modelling of SCOCO and FEZ1 in a heterotetrameric state. Details about the modeling can be found in the supplementary material (Figures S3-S5 and Table S1 in File S1). For the generated model we only imposed the restrains of the interaction between the FEZ monomers as obtained by NMR (Figure 2 and Figure 3). Nonetheless the quality of the obtained model could be validated by three confirmatory facts: 1) the cross-linking, identified by mass spectrometry, between FEZ1-QKEQR^284^ and SCOCO-SKR^81^ maps directly to a region adjacent to the predicted coiled-coil intersections between the two proteins (Figure 1 and Figure 4), 2) Both our SAXS data (Figure 5) as well as data from the literature [40] confirm the stoichiometry of the interaction as 2:2 in wich a FEZ1 dimer complexes with two SCOCO molecules simultaneously (Figure S3 in File S1), and 3) In a previous paper we identified an inter-chain disulfide bond involving the two Cys133 between the two monomers of FEZ1 [18] and in the present model the two cysteines are close to engage into disulfide bond formation.

## Discussion

Transport of vesicles and organelles often occur over long distances. For example, membrane receptors destined for synapses in neuronal cells need to be transported from the cell body down axons that can reach a meter in length. Diffusion would be prohibitively slow and cells have therefore evolved molecular motors that transport vesicle cargoes along microtubule tracks. Kinesins are among these motors and use the energy of ATP to move towards the plus ends of microtubules [4-6]. A general picture is emerging whereby kinesin family members use adaptor/scaffolding proteins to link to their cargoes, and examples of direct interactions with transmembrane proteins exist.

Recent findings propose that FEZ1 could act as a bivalent adaptor of transport mediated by kinesin. As an adapter molecule, the protein FEZ1 recruits both effector and regulatory elements to the processes of formation and transport of vesicles. In fact, in drosophila, UNC-76 protein is involved in kinesin-1 based transport of synaptic precursor vesicle, interacting with synaptotagmin when UNC-76 is phosphorylated by UNC-51 [16], as so in humans, interacting with syntaxin 1 [41]. Futhermore, FEZ1-SCOCO complex is involved in axon outgrowth and vesicle transport [10] and this complex is a likely target for ULK1 (which interacts directly with FEZ1 [14]) regulating the autophagy process [40].

Through its function as adaptor protein, FEZ1 would be able to interact with two other protein molecules simultaneously. The dimeric conformation of FEZ1 by the N-terminal described by Lanza and colleagues (2008) [17] is in accordance with this requirement, since, being a dimer, the protein has two C- terminal regions (involved in protein-protein interaction). This paper sheds light on structural details to this hypothesis, highlighting the interaction of FEZ1 with SCOCO.

The NMR data are in good agreement with the suggested function of FEZ1 as a bivalent transport adaptor and also confirms the covalent disulfide bond between two FEZ1 monomers in a probable anti-parallel topology. *In vivo*, the covalent bond may be required to stabilize the dimer to stand the strong forces involved in the transport of large protein complexes or even organelles. In 2010, we demonstrated by native gel, SAXS and mass spectrometry that FEZ1 dimerizes by disulfide trough the cysteines 133 of each monomer [18]. *In vivo*, this dimeric state covalently bound may be important for transport of proteins mediated by kinesins along microtubules, providing stability to the dimer during the transport process. Furthermore, the protein SCOCO interacts with proteins involved in trans-Golgi network, such as Arl1, present on the membranes of the trans side of the Golgi, probably involved in the recruitment of certain effectors to a specific membrane in the cell. Our data suggest that FEZ1 acts as a link, as an adapter, of the transport machinery mediated by kinesins and proteins from trans-Golgi network destined to vesicle transport, for instance.

In fact, mutations in both FEZ1 and SCOCO cause similar phenotypes and lead to axonal growth deficiencies, mistakes of synaptic vesicles transport, among others. Certainly in the mutant *e11* the interaction with FEZ1/UNC-76 and SCOCO/UNC-69 is disrupted. The mutation promotes the formation of a premature stop codon before the minimal interaction region of SCOCO in FEZ1. The mutant *e587* also has a mutation that generates a premature stop codon, but in SCOCO/UNC-69. However, the truncated region, apparently, does not lose a segment of its coiled-coil (structure involved in protein-protein interaction). The deleted region is predicted to be a random coil (Figure 1), but this region is modeled as an alpha-helix (Figure S2 in File S1). Our results showed that FEZ1 provides possible contacts with SCOCO in this region, but as shown by two-hybrid assay these contacts are not essential for the interaction. Although the same phenotype was observed for the *unc76*/*FEZ1* mutation, in this case the molecular cause seems to be the lack of interaction with its constitutive interaction partner UNC-69/SCOCO (due to the lack of UNC-76 coiled-coil region essential for UNC-69 interaction). Lack of formation of the UNC-76/UNC-69 complex in turn results in the failure of loading the kinesin complex with cargo proteins essential for neuritogenesis.

SAXS experiments confirmed the composition of an heterotetramer of two molecules of 6xHis-FEZ1 (1-392) and 2 molecules of GST-SCOCO (2-82), an interaction partner of FEZ1 identified in two-hybrid screenings in yeast, corroborating the hypothesis of a bivalent adapter. In fact, a recent study showed that after co-transfection of plasmids expressing GFP-FEZ1 and FLAG-SCOCO, a complex of 300 KDa was detected by non-denaturing Blue-native-PAGE, indicating that GFP-FEZ1 and FLAG-SCOCO are also associated in a 2:2 stoichiometry *in vivo* [40].

We believe that the conformation of the modeled complex FEZ1-SCOCO, which was partially confirmed by experimental data coming from different approaches (SAXS, NMR, mass spec.), could represent one of the possible conformations. Certainly, it may not represent its only configuration as it appears under intracellular condition, because we have to bear in mind that FEZ1 has intrinsically unstructured characteristics, that allow it to adopt different conformations, possibly depending on contact with its multitude of different interacting partners other than SCOCO [13,14]. However, the model of the complex FEZ1-SCOCO generated here allowed us to study in detail and with higher confidence, selected regions of interchain contacts between the FEZ1 monomers and between FEZ1 and SCOCO. Interestingly, most regions involved in interchain contact do not appear to be unstructured but to rather adopt alpha-helical secondary elements. This also has been confirmed experimentally by CD spectroscopic studies of the full length and deletion constructs of FEZ1, that showed a significant content of alpha helices, especially in the C-terminal region [17].

The combined features of FEZ1: high content of intrinsically unstructured regions, dimer formation and promiscuity in relation to its large number of interactions with its C-terminal regions as well as its interaction with cytoskeletal elements (kinesin, tubulin, CLASP2), can explain the formation of bridges between microtubules and constriction of the nucleus, resulting in turn in the flower-like phenotype when FEZ1 is over-expressed [42]. The flower-like nuclei are widely observed in leukemia and this feature is considered a morphological hallmark for malign T lymphocytes, which can be observed in more than 50% of the patients with Adult T Cell Leukemia/lymphoma (ATLL) [43-45]. Nuclear contraction and the generation of the multi-lobulated nuclei are related to the forming of tubulin and actin “loops” and involve abnormal microtubule functions [42]. In this case, the covalent dimerization can also be important for stabilization during the of traction forces necessary to constrict the nucleus.

Furthermore, four mood stabilizers (lithium, valproic acid, carbamazepine, and lamotrigine) commonly induce FEZ1 expression in human astrocytes [46]. Through expression data and polymorphisms, FEZ1 has been related to the etiology of schizophrenia and also interacts with the DISC1 protein - one of the most studied genes in this disease. *FEZ1* knockout mice exhibited behavioral phenotypes consistent with the disease. The etiology of schizophrenia is poorly understood, but has been related to signaling mediated by glutamate and dopamine [47]. It was demonstrated that DISC1 regulates the primary cilium, where specific dopamine receptors are located [48]. It is known that trans-membrane proteins are synthesized in the rough endoplasmic reticulum, modified in the Golgi complex and transported through vesicular systems at the surface of the neurons where they are expressed, through Trans-Golgi Network (TGN). Conventional kinesin is involved in transporting vesicles from the TGN to the plasma membrane or endosomes in both neuronal and non-neuronal cells [49]. As a kinesin bivalent adapter, FEZ1 could be involved in the mechanism of vesicle (and consequently membrane receptors) and mitochondria transport during neuronal differentiation, interacting with both cytoskeletal elements and other scaffold proteins, like SCOCO.

In summary, we show here for the first time detailed structural studies on the FEZ1/SCOCO complex as a constitutive kinesis adaptor complex of neurons. The correct loading of kinesin machinery with cargo proteins may resemble a “bar-coding system” which depends on the correct interplay between the double-adaptor FEZ1/SCOCO and their respective protein interaction partners, which are essential for the axon outgrowth and autophagy. Future studies involving deletions and point mutations of both the “double adaptor components” (FEZ1/SCOCO) on the one hand and the cargo proteins involved on the other should help to decode what determines the composition of this supra-molecular complex.

## Supporting Information

File S1 **A single combined File S1 contains supplementary experimental procedures, five Figures S1 to S5, and Table S1.** Figure S1 in File S1. Interaction between FEZ1 and SCOCO in an independent experiment. Purified recombinant proteins FEZ1 and SCOCO were incubated, chemically cross-linked, digested with trypsin, and analyzed by MS. MS/MS spectra were manually validated for b and y ion series of the α (peptide of FEZ1) and β (peptide of SCOCO) chains. The same peptides were found in two independent experiments. Figure S2 in File S1. Assay for β-galactosidase activity in yeast cells shows that FEZ1 (221-392) interacts with the coiled-coil region of SCOCO (2-82 and 2-65) and no interaction was detected with the empty vector pBTM116 (0). In the model, FEZ1 is shown in green and SCOCO in deep blue. The SCOCO region used in the two-hybrid assay is shown in light blue. The highlighted helix in cyan corresponds to the minimal interaction region in FEZ1/UNC-76 with SCOCO/UNC-69. Figure S3 in File S1. FEZ1-SCOCO docking. Relationship between binding energy and cross-linked lysine pair distance between FEZ1 and SCOCO. It was possible to obtain models from the docking routine that exhibit both optimal Cα-Cα distance between lysine pairs (>20 Å) and favorable binding energy (A). (B) General model of FEZ1-SCOCO in heterotetrameric fashion (ratio 2:2). FEZ1 is colored in green and SCOCO in deep blue. Figure S4 in File S1. Profile alignment of 20 templates for FEZ1 protein. Figure S5 in File S1. Per residue Z-score of FEZ1 protein modeling. Table S1 in File S1. Modeled loops in FEZ1 protein.
(PDF)
