# Supplementary material for: Structural Analysis of Intermolecular Interactions in the Kinesin Adaptor Complex Fasciculation and Elongation Protein Zeta 1/ Short Coiled-Coil Protein (FEZ1/SCOCO)
Source: PLoS One. 2013 Oct 7;8(10):e76602. doi: 10.1371/journal.pone.0076602 (PMC3792052; doi:10.1371/journal.pone.0076602)
Supplement: File S1 — A single combined File S1 contains supplementary experimental procedures, five Figures S1 to S5, and Table S1. Figure S1 in File S1. Interaction between FEZ1 and SCOCO in an independent experiment. Purified recombinant proteins FEZ1 and SCOCO were incubated, chemically cross-linked, digested with trypsin, and analyzed by MS. MS/MS spectra were manually validated for b and y ion series of the α (peptide of FEZ1) and β (peptide of SCOCO) chains. The same peptides were found in two independent experiments. Figure S2 in File S1. Assay for β-galactosidase activity in yeast cells shows that FEZ1 (221-392) interacts with the coiled-coil region of SCOCO (2-82 and 2-65) and no interaction was detected with the empty vector pBTM116 (0). In the model, FEZ1 is shown in green and SCOCO in deep blue. The SCOCO region used in the two-hybrid assay is shown in light blue. The highlighted helix in cyan corresponds to the minimal interaction region in FEZ1/UNC-76 with SCOCO/UNC-69. Figure S3 in File S1. FEZ1-SCOCO docking. Relationship between binding energy and cross-linked lysine pair distance between FEZ1 and SCOCO. It was possible to obtain models from the docking routine that exhibit both optimal Cα-Cα distance between lysine pairs (>20 Å) and favorable binding energy (A). (B) General model of FEZ1-SCOCO in heterotetrameric fashion (ratio 2:2). FEZ1 is colored in green and SCOCO in deep blue. Figure S4 in File S1. Profile alignment of 20 templates for FEZ1 protein. Figure S5 in File S1. Per residue Z-score of FEZ1 protein modeling. Table S1 in File S1. Modeled loops in FEZ1 protein. (PDF) [file pone.0076602.s001.pdf]

SUPPLEMENTARY EXPERIMENTAL PROCEDURES

Yeast two-hybrid assay

The yeast two hybrid assay was performed according to described in Assmann (2006) [01] and was performed with the baits SCOCO (2-82) and SCOCO (2-65) and prey FEZ1 (221-392).

In silico modeling

According to the YASARA homology modeling routine, an alignment with the target sequence is obtained using different sources of information such as: sequence-based profiles and structure-based profiles for both the target and the template. A structure-based alignment correction, based on SSALN matrices [02], considers structural information in order to avoid gaps in secondary structure elements [03]. The presented alignment (Supplementary Figure 4), in which 20 of the 47 template profile alignments are displayed, 227 of 392 target residues accounting for 57.9% its total sequence were aligned to template residues. Amongst these residues, sequence identity if 18.9% and 39.9% are similar, BLOSUM62 score > 0 and 16 loops had to be modeled (Supplementary Table 1). The final model based on the template with pdb id 1XD4 had a Z-Score of 0.366 for dihedral, -3.052 for Packing 1D, -1.743 for Packing 3D and an overall of -1.947, thus being a satisfactory model. Per residue Z-score can be seen on Supplementary Figure 5.

SUPPLEMENTARY REFERENCES

[01] Assmann EM, Alborghetti MR, Camargo MER, Kobarg J (2006) FEZ1 dimerization and interaction with transcription regulatory proteins involves its coiled-coil region. J Biol Chem 281: 9869–9881. doi:10.1074/jbc.M513280200.

[02] Qiu J and Elber R (2006) SSALN: An alignment algorithm using structure-dependent substitution matrices and gap penalties learned from structurally aligned protein pairs Proteins 62,881-891

[03] King RD and Sternberg MJE (1996) Identification and application of the concepts important for accurate and reliable protein secondary structure prediction. Protein Sci. 5,2298-2310

SUPPLEMENTARY FIGURES

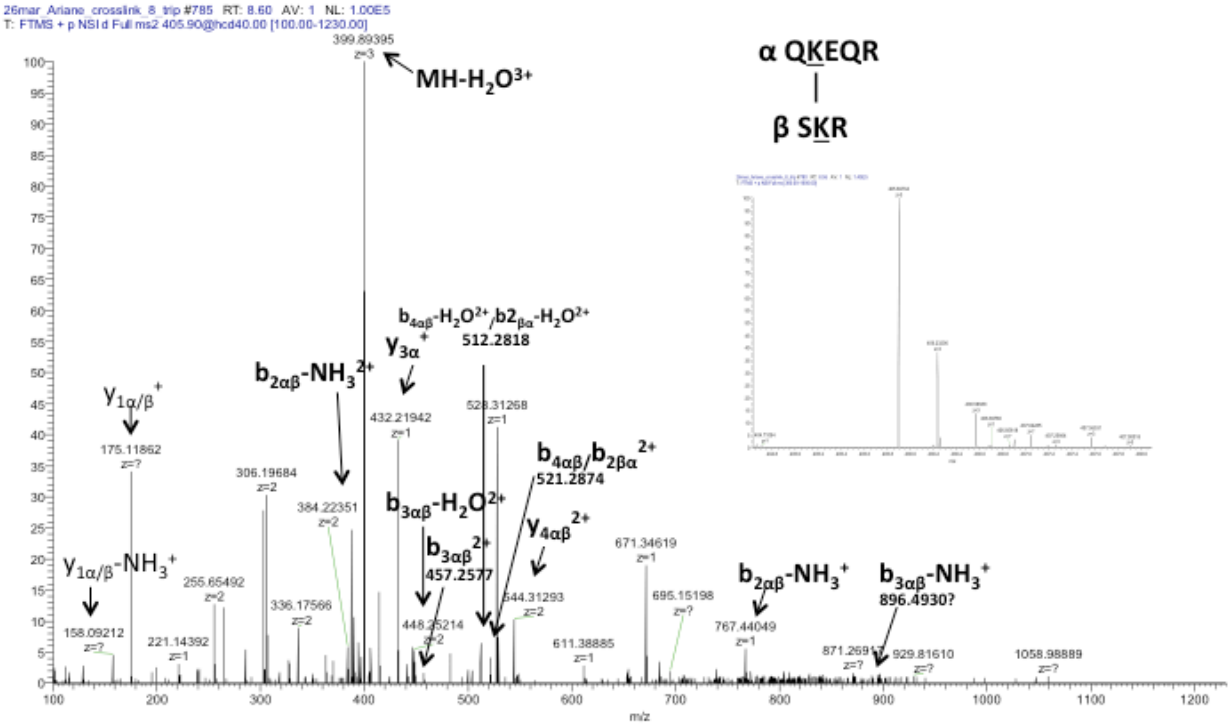

SUPPLEMENTARY FIGURE 1. Interaction between FEZ1 and SCOCO in an independent experiment A) Purified recombinant proteins FEZ1 and SCOCO were incubated, chemically cross-linked, digested with trypsin, and analyzed by MS. MS/MS spectra were manually validated for b and y ion series of the α (peptide of FEZ1) and β (peptide of SCOCO) chains. The same peptides were found in two independent experiments.

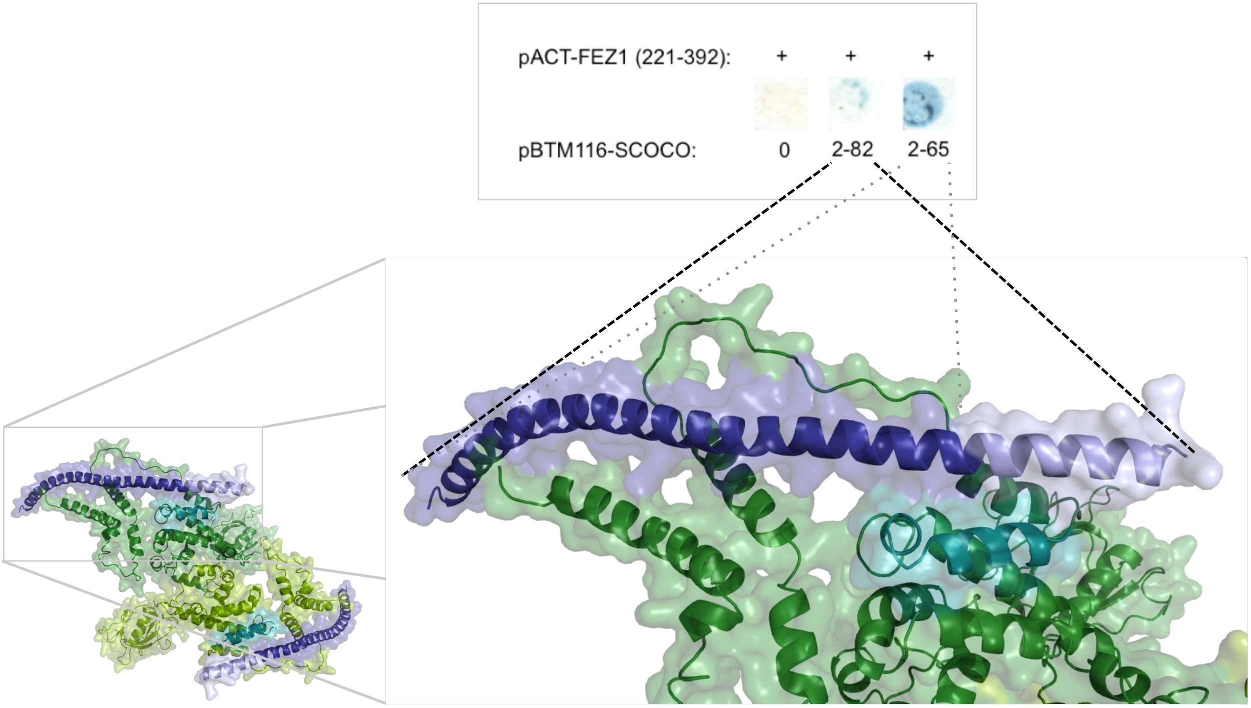

SUPPLEMENTARY FIGURE 2. Assay for *β*-galactosidase activity in yeast cells shows that FEZ1 (221-392) interacts with the coiled-coil region of SCOCO (2-82 and 2-65) and no interaction was detected with the empty vector pBTM116 (0). In the model, FEZ1 is shown in green and SCOCO in deep blue. The SCOCO region used in the two-hybrid assay is shown in light blue. The highlighted helix in cyan corresponds to the minimal interaction region in FEZ1/UNC-76 with SCOCO/UNC-69.

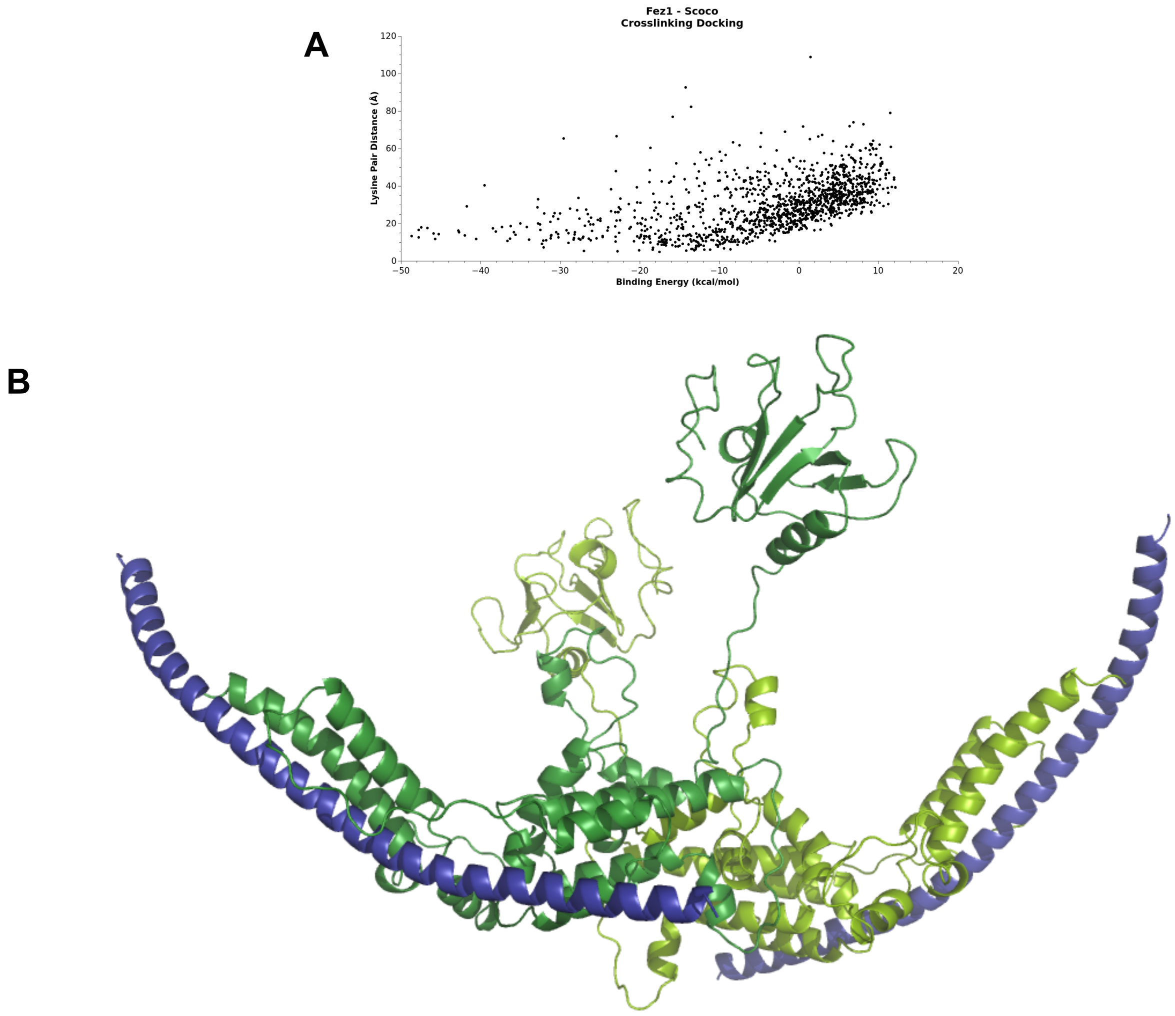

SUPPLEMENTARY FIGURE 3. A) Relationship between binding energy and cross-linked lysine pair distance between FEZ1 and SCOCO. It was possible to obtain models from the docking routine that exhibit both optimal Cα-Cα distance between lysine pairs (>20 Å) and favorable binding energy. B) General model of FEZ1-SCOCO in heterotetrameric fashion (ratio 2:2). FEZ1 is colored in green and SCOCO in deep blue.

```
Q59EU2  : ..................................................................EEKLSLCFR..........PPRTAVRPITERSLLQGDEIWNA (AA)
C3XZ65  : MAAPLAQIDDEWLD............................VDNVDNCGFGVVSFKSMEDLVHEFDETLNICFRNYNAKTDSIAPV.........EEIMENSELWQG (AB)
UPI00020: ............................................................................................................ (AC)
UPI00019: ........................................................................................................IWNA (AD)
UPI00016: ..................................................AHVVALTCAEDAAKHLDETISVCFRSVHARTDTADPV..........SLLEDDEVWIA (AE)
E0CYY9  : MEAPLVSLDEEFEDIRPSCTEEPEEKPQCLYGTSPHHLEDPSLSELENFSSEIISFKSMEDLVNEFDEKLNVCFRNYNAKTESLAPVKNQLQIQEEEETLRDEEVWDA (AF)
UPI00020: .......................................................FKSMEDLVNEFDEKLSVCFRNYSTDTGTIAPV............MKDDEIWNA (AG)
E1C355  : MEAPLVSLEEEF...................GGVPRRTMDPALAELESFSTEMMSFKSMEDLVQEFDEKLTVCFRNYDATTEGLAPVRGRLQAQEEEEHLQDEEVWDA (AH)
Q6PBT3  : MEAPLVCLDEEFEDLRPCKMEELEEQPPC.............FSELENF..EMMSFKSMEDLVNEFDEKLNVCFHNYNTKTEGLAPVRNQSHAEEDEERLQDEDVWDA (AI)
Q99689  : MEAPLVSLDEEFEDLRPSCSEDPEEKPQCFYGSSPHHLEDPSLSELENFSSEIISFKSMEDLVNEFDEKLNVCFRNYNAKTENLAPVKNQLQIQEEEETLQDEEVWDA (AJ)
SecStr  : CCCCCCCCCCCCCCCCCCCCCCCCCCCCCCCCCCCCCCCCCCCHHCCCCCHHHHCCCCHHHHHHHHHHHHHHHCCCCCCCCCCCCCCCCCCCCHHHHHHHHHHHHHHH (AK)
Target  : MEAPLVSLDEEFEDLRPSCSEDPEEKPQCFYGSSPHHLEDPSLSELENFSSEIISFKSMEDLVNEFDEKLNVCFRNYNAKTENLAPVKNQLQIQEEEETLQDEEVWDA
Match   :        L E:F                         L:D            I:S KS E|  N :   |:: |R                   :  E | D  : :
Template: ....EYRLKEKFF.......................ILKDE..........VIFSAKSAEEKNNWMAALISLQYR...................STLERMLDVTMLQE
SecStr  : ....EEEEEEEEC.......................ETTTT..........EEEEECHHHHHHHHHHHHHHHHHC...................HHHHHHHHHHHHHH (AL)
UPI00015: ....EYRLKEKFF.......................ILKDE..........VIFSAKSAEEKNNWMAALISLQYR...................STLERMLDVTMLQE (AM)
UPI00004: ....EYRLKEKFF.......................ILKDE..........VIFAAKSAEEKNNWMAALISLQYR...................STLERMLDVTMSQE (AN)
UPI00016: ....EYRLKEKFF.......................ILKDG..........VVFAAKSAEEKNSWMAALISLQYH...................STLERMLDSALLRE (AO)
F1N8Q9  : ....EYRLKEKII.......................VSKDE..........ILFAAKSAEEKSNWMAALISLQYR...................STLDRMLDSVLLQE (AP)
Q07890  : ....EYRLKEKFV.......................VSKDE..........IIFAAKSAEEKNNWMAALISLHYR...................STLDRMLDSVLLKE (AQ)
UPI00020: ....EYRLKEKIV.......................VPKDE..........FIFAAKSAEEKNNWMAVLISLQYR...................STLDRMLDAVLLQE (AR)
F1R019  : ....EFRLKEKFV.......................VGREE..........AVFSARSAEEKSAWMAALVTLQYR...................PTLDRMLDTVLHRE (AS)
UPI00016: ....EYRLKEKFV.......................IGKDE..........AVFCARTAEEKAAWMAALVTLQYR...................STLDRMLDTVLQHE (AT)
Q4T188  : ....EYRLKEKFF.......................ILKDG..........VVFAAKSAEEKNSWMAALISLQYH...................STLERMLDSAMLRE (AU)
UPI0001E: ....EYRLKEKFF.......................ILKDE..........VIFSAKSAEEKNNWMAALISLQYR...................STLERMLDVTMLQE (AV)
C3XZI5  : ....EYRLKETFF.......................VARDQ..........TIFFAKNEDEKNNWMAALITLHCR...................STLERMLDSILLEE (AW)
UPI0000E: ....EYRFKERYY.......................EVKDS..........IVFFAKSAEEKTSWMEALITLLHR...................STLERMLDTILQQE (AX)
F4WM45  : ....ELRLKERLF.......................APRDH..........VILVAKSVEDKNNWMADLVMLNTK...................SMLERTLDSILLDE (AY)
E9H784  : ....DYRLKEKFF.......................APRQQ..........CVLFTRTAEEKANWMAALVMLNTK...................SMLERTLDVILLDE (AZ)
D2A5R8  : ....ECRLKERFF.......................APRHQ..........VILCAKTAEEKNSWMADLVMLNTR...................SMLERILDSILSDI (BA)
UPI00017: ....KFKLKEKLF.......................FPRLE..........ITLVARTFEDKANWMADLLMLTSK...................SILDRTLNNILLDE (BB)
E2AK89  : ....ELRLKERFF.......................APRDH..........VILVAKSVEDKNNWMADLVMLNTK...................SMLERTLDSILLDE (BC)
UPI0001C: ....................................SVKDQ..........TVFFAKSAEDKANWMAVLTALHYR...................STLERMLDTILTDE (BD)
Q9N5D3  : .....LKFKDRLA.......................ESHDK..........LHFVCKNPEEKRQWMAVLVKVTTK...................SVLDRILDNHEKEE (BE)
E5SJ49  : ....................................RQRDQ..........IVLLAADPDEKCTWLAALLMLQTR...................SMLERMLDSHLEAE (BF)
```

```
Q59EU2  : LTDNYGNVMPVDW...................................NDEPLFTADQVIEEIEEMMQESPDPEDDE..........TQSDRLSMLSQEIQTLKR... (AA)
C3XZ65  : LTDNFANVLPVDW.................................SLTQEPIFTAEEVIEEIDEIM..........................SVLSQEMLALRERSN (AB)
UPI00020: ..........................................................VIEEIEEMMQESPDP..............TQSDRLSMLSQEIQTLKRSST (AC)
UPI00019: LTDNYGNVMPVDWKSSHTRA..............DEELREQ.......NEEPLFTAEQVIEEIEEMMQESPDPED............TQSDRLSILSQEIQTLKRSST (AD)
UPI00016: LTSNYGLVRPVDWKQ................PSDEEELREQLDM.....EEPLLTAEQVIEEIEEIMQDSP..................................... (AE)
E0CYY9  : LTDNYIPSLSEDWRDPNIEALNGNSSDIEIHEKEEEEFNEKSENDSGINEEPLLTADQV................................................. (AF)
UPI00020: LTDNYGNVMPVDWKTSHARAL..NLLDKAVNE.DDEELREQLD.....NEEPLFTAEQVIEEIEEMMQESPDP....................SMLSQEIQTL..... (AG)
E1C355  : LTDGFVP.....WMHPEAEAPDG...DPQLCEKEDEELAERSEHDSGINEEPLLTAEQVIEELEELMQSSPDP.DEEEEEDEEEDAEANAEGDS.VLHELHTFSPTFN (AH)
Q6PBT3  : LTDNYMPSSVSSWDDPTSETLNGNLSDQEIHEKDEDEMNEKNENANCLNEEPLITADQVIEEIVEMMENSPDPGETEE..EEDDIGVSSSKSSPSLLEEIRMLSQASN (AI)
Q99689  : LTDNYIPSLSEDWRDPNIEALNGNCSDTEIHEKEEEEFNEKSENDSGINEEPLLTADQVIEEIEEMMQNSPDPEEEEEVLEEEDGGETSSQADSVLLQEMQALTQTFN (AJ)
SecStr  : HHCCCCCCCCCCCCCCCCCCCCCCCCCHHHHHHHHHHHHHHCCCCCCCCCCCCCCHHHHHHHHHHHHHCCCCCCCHHHHHHHHCCCCCCCCCCHHHHHHHHHHHHCCC (AK)
Target  : LTDNYIPSLSEDWRDPNIEALNGNCSDTEIHEKEEEEFNEKSENDSGINEEPLLTADQVIEEIEEMMQNSPDPEEEEEVLEEEDGGETSSQADSVLLQEMQALTQTFN
Match   :                          S| :I  :E                 P|| A  VI: IE:|     DP:  :  L    :    :|  S:|||::|
Template: ........................DSEENIIFEE................IPIIKAGTVIKLIERL....ADPNFVRTFLTTYRSFCKPQELLSLIIERFE.......
SecStr  : ........................CTTTTCEECC................CCEEEECHHHHHHHHH....CCHHHHHHHHHTTTTCCHHHHHHHHHHHHHH....... (AL)
UPI00015: ........................DSEENIVFEE................IPIIKAGTVIKLIERL....ADPNFVRTFLTTYRSFCKPQELLSLLIGRFE....... (AM)
UPI00004: ........................DSEENIVFEE................IPIIKAGTVVKLIERL....ADPNFVRTFLTTYRSFCKPQELLSLLIERFE....... (AN)
UPI00016: ........................DSEENVVFED................IPIIKAGTVLKLIERL....ADPNFVRTFLTTYRSFCKPQELLDLLMERFE....... (AO)
F1N8Q9  : ........................DSEDNIVFED................IPIIKGGTVVKLIERL....ADPNFVRTFLTTYRSFCKPQELLSLLIERFE....... (AP)
Q07890  : ........................DSEENIVFED................IPIIKGGTVVKLIERL....ADPNFVRTFLTTYRSFCKPQELLSLLIERFE....... (AQ)
UPI00020: ........................DSEENIVFED................IPLIKGGTVVKLIERL....ADPNFVRTFLTTYRSFCKPQELLSLLIERFE....... (AR)
F1R019  : ........................DSEENIVFEE................IPIIKAGTVVKLIERL....ADPNFVRTFLTTYRSFCKPQDLLTLLIERIE....... (AS)
UPI00016: ........................DSEENIVFED................IPIIKAGTVVKLIERL....ADPNFVRTFLTTYRSFCKPQELLTLLIERIE....... (AT)
Q4T188  : ........................DSEENVVFEE................IPIIKAGTVLKLIERL....ADPNFVRTFLTTYRSFCKPQELLELLMERFE....... (AU)
UPI0001E: ........................DSEENIIFEE................IPIIKAGTVIKLIERL....ADPNFVRTFLTTYRSFCKPQELLSLIIERFE....... (AV)
C3XZI5  : ........................DRPDNIVFED................VPVIKGGSLIKLIERL....ADPTFVRTFLTTYRSISKPCELLDLLIERFE....... (AW)
UPI0000E: ........................DSPDNIVFEE................VPLIKGGTLFKLIERL....ADPSFVRTFLTTYRSFCSPQDLLSLLFKRFE....... (AX)
F4WM45  : ........................DSPENIVLEA................VPLIKGATLIKLVERL....ADPAFVRTFLTTYRSFCSPQELLALLIERFD....... (AY)
E9H784  : ........................DSETNLVLET................VPLIKGATLLKLIERL....ADPMLVRTFLTTYRSFCSPTELLELLIERFE....... (AZ)
D2A5R8  : ........................DSKDNIILEQ................VPLIKGATLYKLVERL....ADPKFVRTFLTTYRSFCSPKELLDLLIERFQ....... (BA)
UPI00017: ........................DSRSNIIFEE................IPLIKGAILLKLIERL....ADPKFVKTFLTIYRSFCSPHDLMDLLIERYK....... (BB)
E2AK89  : ........................DSKENIVLEA................VPLIKGATLIKLVERL....ADPAFVRTFLTTYRSFCSPQELLTLLIERFD....... (BC)
UPI0001C: ........................DSEDNIVFED................IQVIRGGSLLKLIERL....ANPSFVRTFLTTYRSFSSPYIMCNNSLRTFE....... (BD)
Q9N5D3  : ........................DTEDNISFED................IPVIKCGTVLKLIERL....TDSKYILTFLISYRSFCTPNDLFSLLLERFN....... (BE)
E5SJ49  : ........................DSEDNIVLED................TS..SSGIPVLLIERF....PDPS..........AGTMAAALLS.............. (BF)
```

SUPPLEMENTARY FIGURE 4 (part 1/3). Profile alignment of 20 templates for FEZ1 protein.

```
Q59EU2  : .......VKRLSVSELNEILEEIETAIKEYSEELVQQLALRDELEFEKEVKNSFISVLIEVQNKQKEHKETAKKKKKLKNGSSQNGKNER.................. (AA)
C3XZ65  : TVTSY..LKKLPLQTLNEVLEELEATIKEYSEILIQQLALRDELVYEKEVKNQFISALVAVQNRQREHKQNKDNKKKRK............................. (AB)
UPI00020: NNSYEERVKRLSVAELNELLEEIETAIKDYSEELVQQLALRDELEFEKEVKNSFISVLIEVQNKQREHKETAKKKKKLKNGSPQNGKQERG..MPGTRFSMEGISNVI (AC)
UPI00019: NNSYEERVKRLSVAELNELLEEIETAIKDYSEELVQQLALRDELEFEKEVKNSFISVLIEVQNKQREHKETAKKKKKLKNGSPQNGKQERG..MPGTRFSMEGISNVI (AD)
UPI00016: .......LRALSVANLNEHLEGTETDIRRFSEELVQQLALRDELDFEKEVKNTFISTLIDVQNRQKEQRELMKRKRSDGATGASASHISSLSFSPPQRFSMEALSSAI (AE)
E0CYY9  : ............................................................................................................ (AF)
UPI00020: .......VKMMSVAELNDLLEEIETAIKDYSEELIQQLALRDELEFEKEVKNSFISVLIEVQNKQKEHKENVKKKRRVKSANAQNGTKE....MPGTRFSMEGLSNVI (AG)
E1C355  : NNCSHEGLEQLTAGELAAAAGRAEAASRALSAELVAQLARRDELAFEKEVKTAFIGALLAVQGEQREQREAARRRRRDRGLSMQGGRPERGGHMPRKRFSMEGISSIL (AH)
Q6PBT3  : NNCSYEGLRLMPSSALLDILCRMEAAIREYSEELVAQLARRDELEFEKEVKNTFITALMEVQNRQKEQRELSKRRRKDKAMSL...RPEKTGSMPAKRFSMEGLSNIL (AI)
Q99689  : NNWSYEGLRHMSGSELTELLDQVEGAIRDFSEELVQQLARRDELEFEKEVKNSFITVLIEVQNKQKEQRELMKKRRKEKGLSLQSSRIEKGNQMPLKRFSMEGISNIL (AJ)
SecStr  : CCCCHHHHCCCCHHHHHHHHHHHHHHHHHHHHHHHHHHHHHHHHHHHHHHHHHHHHHHHHHHHHHHHHHHHHHHHHCCCCCCCCCCCCCCCCCCCCCCCCCCCCHHHH (AK)
Target  : NNWSYEGLRHMSGSELTELLDQVEGAIRDFSEELVQQLARRDELEFEKEVKNSFITVLIEVQNKQKEQRELMKKRRKEKGLSLQSSRIEKGNQMPLKRFSMEGISNIL
Match   : :: :  :|:   ::EL ::  |    |:    :: :: :::   |FE  : : :   |  :::  ::  :|::|||      :QSS
Template: TEADRIAIE...SAELKRFRKEYIQPVQLRVLNVCRHWVEHHFYDFEAYLLQRMEEFIGTMKKWVESITKIIQRKKI.....FQSSP.....................
SecStr  : HHHHHHHHH...HHHHHHHHHCHHHHHHHHHHHHHHHHHHHHHHCHHHHHHHHHHHHHHHHHHHHHHHHHHHHHHHH.....CCCCC..................... (AL)
UPI00015: TEADRRAME...SAELKRFRKEYIQPVQLRVLNVCRHWVEHHFYDFEADLLQRLEEFIGTMKKWVESITKIIQRKKQ.....FESSP..................... (AM)
UPI00004: SEADRIAME...SAELKRFRKEYVQPVQLRVLNVCRHWVEHHFYDFESDLLIRLEEFIGTMKKWVESITKIINRKKP.....FESLP..................... (AN)
UPI00016: SELDQL..E...SPEVKRFRKEFVQPVQLRVLNVCRHWVEHHFYDFEPQLLRTLEEFISSMRKWVESITKIIQRKKP.....FQNSP..................... (AO)
F1N8Q9  : TEADRLAIE...SADLKRFRKEYVQPVQLRILNVFRHWVEHHFYDFELELLERLETFISSMKKWVESIAKIIKRKKI.....FESPP..................... (AP)
Q07890  : TDADKLAIE...SADLKRFRKEYVQPVQLRILNVFRHWVEHHFYDFELELLERLESFISSMKKWVESIAKIIRRKKV.....FESPP..................... (AQ)
UPI00020: TEADRLAIE...SADLKRFRKEYVQPVQLRILNVFRHWVEHHYYDFEQELLDRLETFISTMKKWVESIAKIIRRKKV.....FESPP..................... (AR)
F1R019  : SEADREALW...AAELQRFRREYVQPVQLRVLNVFRQWVEHHFYDFEPELYERLEGYLTQMRKWVESISKIMRRKGI.....FESPP..................... (AS)
UPI00016: TEEDRQALW...AAELQRFRKEYVQPVQLRVLNVFRQWVEHHFYDFEPELRSHLEEYITSMRKWVESINKIIKRKGV.....FESPP..................... (AT)
Q4T188  : SELDQM..E...SPEVKRFRKEFVQPVQLRVLNVCRHWVEHHFYDFEPNLLRTLEEFIASMRKWVESITKIIQRKKP.....FQNSP..................... (AU)
UPI0001E: TEADRIAIE...SAELKRFRKEYIQPVQLRPPNLCRH....PYYDFEADLLQQMEXFIVIMKKWVESITKIIQRKKI.....FQSSP..................... (AV)
C3XZI5  : TPEDQAAID...REDLKRFRKEYAQPVQLRVLNVFRHWVDHHFYDFEKELLDKLMEFIEGMRKWVDSITKIVLRENV.....FERTP..................... (AW)
UPI0000E: TEEDQASID...REDLKRFRKEYAQPIQLRVLNVFRHWVDQHFYDFESELLGSLEKFIDSMRKWVESIKKIIMRREQ.....FEKDP..................... (AX)
F4WM45  : VYGEEEK.P...REDWKRYRKEFCQPVQFRVLNVLRHWVDHHFYDFERNLLERLQLFLDTMRKWVDSVIKIVQRKQR.....FERSP..................... (AY)
E9H784  : DETSGLGVD...SRELKRFRKQYMQPVQFRVLNVLRHWVDHHFYDFEPGLLQRLQGFLGVVRKWVESIHKIVARREE.....FDRSP..................... (AZ)
D2A5R8  : ECCDTDKMQ...REDWKKYRKEYVQPVQFRVLNVLRHWVDHHFYDFEPTLLVKLRDFLDAMRKWVDSVLKILHRKME.....FDEPP..................... (BA)
UPI00017: ......GIT...RDETKRFKKEYLIPVQFRVLNVFRHWVDYHYYDYQPYLLDKLYEFLDSIKKLTDSLIKIIHRKEA.....FDSPP..................... (BB)
E2AK89  : VYGEEEKPS...REDWKRYRKEFCQPVQFRVLNVLRHWVDHHFYDFERNLLERLQLFLDTMRKWVDSVLKIVQRKQR.....FERSP..................... (BC)
UPI0001C: TEEDESALR...REDLKKFRKEYAQPVQLRVLNVFRHWVDQHFYDFEPELLERLMEFIDNMRKWVESIKKIVQRRSL.....FDQTP..................... (BD)
Q9N5D3  : LQQPKTVQS...SSSSQKFRKEFQQPIQLRVLSVINQWVKLHWYDFQPVLLDALELFLNRHKKFCKTILALIEKRDE.....FQQHP..................... (BE)
E5SJ49  : ENKSNMDVS...RESLKRLRKEYIQPVRIRVLNIIRQWIDHHWYDFDAELLANLKHFLKRVVKWTRSLQNIIDRK............................... (BF)
```

```
Q59EU2  : .................................................................... (AA)
C3XZ65  : .................LYTVIPYEKKNSPMTAEDLQILTKILVAMSEDSDKVPSLLTDYILK..... (AB)
UPI00020: QNGFRHTFGNSGGEKQYLTTVIPYEKKNGPPSVEDLQTLTKILHAMKEDSEKVPSLLTDYILK..... (AC)
UPI00019: QNGFRHTFGNSSGEKQYLTTVIPYEKKNGPPSVEDLQTLTKILHAMKEDSEKVPSLLTDYILKVLCPT (AD)
UPI00016: QSSFRNTFGSSCSEPQYLTTVIPYEKKGRPPSLRDLQILTKILQAMRDDSDKVPGLLTDYILQVLCPT (AE)
E0CYY9  : .................................................................... (AF)
UPI00020: QNSFRQTFGNPGGEKQYLTTVIPYEEKNGPPSIEDLQILTKILRAMKDDSDKVPSLLTDYILKVLCPT (AG)
E1C355  : HSGLRQTFGPAANEKQYLNTVIPYEKKGSPPSVEDLQMLTNILFAMKEGNEKVPTLLTDYILKVLCPT (AH)
Q6PBT3  : QTGIRQTFGSSGTEKQYLNTVIPYEKKGTPPSVDDLQMLTKILYAMKEDSEKVPTLLTDYILKVLCPT (AI)
Q99689  : QSGIRQTFGSSGTDKQYLNTVIPYEKKASPPSVEDLQMLTNILFAMKEDNEKVPTLLTDYILKVLCPT (AJ)
SecStr  : HCCCCCCCCCCCCCCCCCCCCCCCCCCCCCCCHHHHHHHHHHHHHHHHCCCCCCHHHHHHHHHHCCCC (AK)
Target  : QSGIRQTFGSSGTDKQYLNTVIPYEKKASPPSVEDLQMLTNILFAMKEDNEKVPTLLTDYILKVLCPT
Match   :                  L T:        P :|     L :  :     :E V       |LK:|  T
Template: ................DLLTL.......HPIEIARQLTLLESDLYRA..SELVG....PNLLKMIRHT
SecStr  : ................TTTTC.......HHHHHHHHHHHHHHHHHHH..GGTTT....HHHHHHHHHH (AL)
UPI00015: ................DLLTL.......HPIEIARQLTLLESDLYRA..SELVG....PNLLKMIRHT (AM)
UPI00004: ................DLLTL.......HPIEIARQLTLLESDLYRA..SELVG....PNLLKMIRHT (AN)
UPI00016: ................DLMTL.......HPIEIARQLTLLESDFFRA..SELVG....PNLLRMIRHT (AO)
F1N8Q9  : ................DLMTL.......HPIEIARQLTLLESDLYRA..SELVG....PNLLKMIRHT (AP)
Q07890  : ................DLMTL.......HPIEIARQLTLLESDLYRK..SELVG....PNLLKMIRHT (AQ)
UPI00020: ................DLLTL.......HPIEIARQLTLLESDLYRA..SELVG....PNLLKMIRHT (AR)
F1R019  : ................DLMTL.......HPIEIARQLTLLESDLYRA..SELVG....PNLLRMIRHT (AS)
UPI00016: ................DLMTL.......HPIEIARQLTLLESELYRA..SELVG....PNLLRMIRHT (AT)
Q4T188  : ................DLMTL.......HPIEIARQLTLLESDFFRA..SELVG....PNLLRMIRHT (AU)
UPI0001E: ................DLLTL.......HPIEIARQLTLLESDLYRC..Q..............ILHS (AV)
C3XZI5  : ................ELLTL.......HPIEIARQLTLLEFDLYRA..SELVG....PNLLKMIHHS (AW)
UPI0000E: ................DIMTL.......HPIEIARQLTLLESDLYRA..SELVG....PNLLKMIHHS (AX)
F4WM45  : ................GILTL.......HPIELARQLTLLEFELYKT..SELVG....PNLLKMIKHT (AY)
E9H784  : ................DLMTL.......HPIEIARQLTLLEFDLYRA..SELVG....PNLLRMIHHT (AZ)
D2A5R8  : ................GILTL.......HPVEIARQLTILEFDLYRM..SELVG....PNLLKMIKHT (BA)
UPI00017: ................NILLV.......HPLEFARQLTLIESENYRA..SELVG....PNLLRLIKRT (BB)
E2AK89  : ................GILTI.......HPIELARQLTLLEFELYRV..SELVG....PNLMKMIKHT (BC)
UPI0001C: ................DLMTL.......HPVEIARQLTLLEFDLFRA..SELVG....PNLLKMIQQT (BD)
Q9N5D3  : ................DLLTL.......HPIEIGRQLTLLHSDLYRA..IELVE....PQLLRLTDHS (BE)
E5SJ49  : ....................L.......HPIEIARQITLLEFDLYRA..IELVG....PQLLKLIQHS (BF)
```

SUPPLEMENTARY FIGURE 4 (part 2/3). Profile alignment of 20 templates for FEZ1 protein.

SUBTITLES:

(AA) [uniref90|Q59EU2 Fasciculation and elongation protein zeta 2 variant (Fragment) n=5 Tax=Eutheria RepID=Q59EU2_HUMAN]
(AB) [uniref90|C3XZ65 Putative uncharacterized protein (Fragment) n=1 Tax=Branchiostoma floridae RepID=C3XZ65_BRAFL]
(AC) [uniref90|UPI0002034F43 PREDICTED: visual pigment-like receptor peropsin-like n=1 Tax=Meleagris gallopavo RepID=UPI0002034F43]
(AD) [uniref90|UPI000194C19E PREDICTED: similar to zygin 2 n=1 Tax=Taeniopygia guttata RepID=UPI000194C19E]
(AE) [uniref90|UPI00016E8843 UPI00016E8843 related cluster n=1 Tax=Takifugu rubripes RepID=UPI00016E8843]
(AF) [uniref90|E0CYY9 Uncharacterized protein n=3 Tax=Eutheria RepID=E0CYY9_MOUSE]
(AG) [uniref90|UPI0002065F2E UPI0002065F2E related cluster n=1 Tax=Xenopus (Silurana) tropicalis RepID=UPI0002065F2E]
(AH) [uniref90|E1C355 Uncharacterized protein n=2 Tax=Phasianidae RepID=E1C355_CHICK]
(AI) [uniref90|Q6PBT3 Fasciculation and elongation protein zeta 1 (Zygin I) n=1 Tax=Danio rerio RepID=Q6PBT3_DANRE]
(AJ) [uniref90|Q99689 Fasciculation and elongation protein zeta-1 n=18 Tax=Theria RepID=FEZ1_HUMAN]
(AK) [Secondary structure predicted by PsiPred]
(AL) [Secondary structure assigned by YASARA]
(AM) [uniref90|UPI0001554446 PREDICTED: similar to son of sevenless homolog 1 (Drosophila) n=1 Tax=Ornithorhynchus anatinus RepID=UPI0001554446]
(AN) [uniref90|UPI00004D0C97 Son of sevenless homolog 1 (SOS-1). n=2 Tax=Xenopus (Silurana) tropicalis RepID=UPI00004D0C97]
(AO) [uniref90|UPI00016E51B5 UPI00016E51B5 related cluster n=7 Tax=Tetraodontidae RepID=UPI00016E51B5]
(AP) [uniref90|F1N8Q9 Uncharacterized protein (Fragment) n=4 Tax=Amniota RepID=F1N8Q9_CHICK]
(AQ) [uniref90|Q07890 Son of sevenless homolog 2 n=25 Tax=Mammalia RepID=SOS2_HUMAN]
(AR) [uniref90|UPI000203AE7E PREDICTED: son of sevenless homolog 2-like n=1 Tax=Anolis carolinensis RepID=UPI000203AE7E]
(AS) [uniref90|F1R019 Uncharacterized protein (Fragment) n=5 Tax=Danio rerio RepID=F1R019_DANRE]
(AT) [uniref90|UPI00016E23BC UPI00016E23BC related cluster n=4 Tax=Tetraodontidae RepID=UPI00016E23BC]
(AU) [uniref90|Q4T188 Chromosome undetermined SCAF10698, whole genome shotgun sequence n=1 Tax=Tetraodon nigroviridis RepID=Q4T188_TETNG]
(AV) [uniref90|UPI0001E86FA2 PREDICTED: LOW QUALITY PROTEIN: son of sevenless homolog 1-like n=1 Tax=Sus scrofa RepID=UPI0001E86FA2]
(AW) [uniref90|C3XZI5 Putative uncharacterized protein (Fragment) n=1 Tax=Branchiostoma floridae RepID=C3XZI5_BRAFL]
(AX) [uniref90|UPI0000E471A9 PREDICTED: similar to Son of sevenless homolog 2 (Drosophila) n=1 Tax=Strongylocentrotus purpuratus RepID=UPI0000E471A9]
(AY) [uniref90|F4WM45 Protein son of sevenless n=1 Tax=Acromyrmex echinatior RepID=F4WM45_9HYME]
(AZ) [uniref90|E9H784 Putative uncharacterized protein n=1 Tax=Daphnia pulex RepID=E9H784_DAPPU]
(BA) [uniref90|D2A5R8 Histone H2A n=2 Tax=Tribolium castaneum RepID=D2A5R8_TRICA]
(BB) [uniref90|UPI0001791FA8 PREDICTED: protein son of sevenless-like n=1 Tax=Acyrthosiphon pisum RepID=UPI0001791FA8]
(BC) [uniref90|E2AK89 Protein son of sevenless n=1 Tax=Camponotus floridanus RepID=E2AK89_9HYME]
(BD) [uniref90|UPI0001CBABEA PREDICTED: son of sevenless homolog 1-like n=1 Tax=Saccoglossus kowalevskii RepID=UPI0001CBABEA]
(BE) [uniref90|Q9N5D3 Drosophila sos homolog protein 1 n=2 Tax=Caenorhabditis elegans RepID=Q9N5D3_CAEEL]
(BF) [uniref90|E5SJ49 Protein son of sevenless n=1 Tax=Trichinella spiralis RepID=E5SJ49_TRISP]

SUPPLEMENTARY FIGURE 4 (part 3/3). Profile alignment of 20 templates for FEZ1 protein.

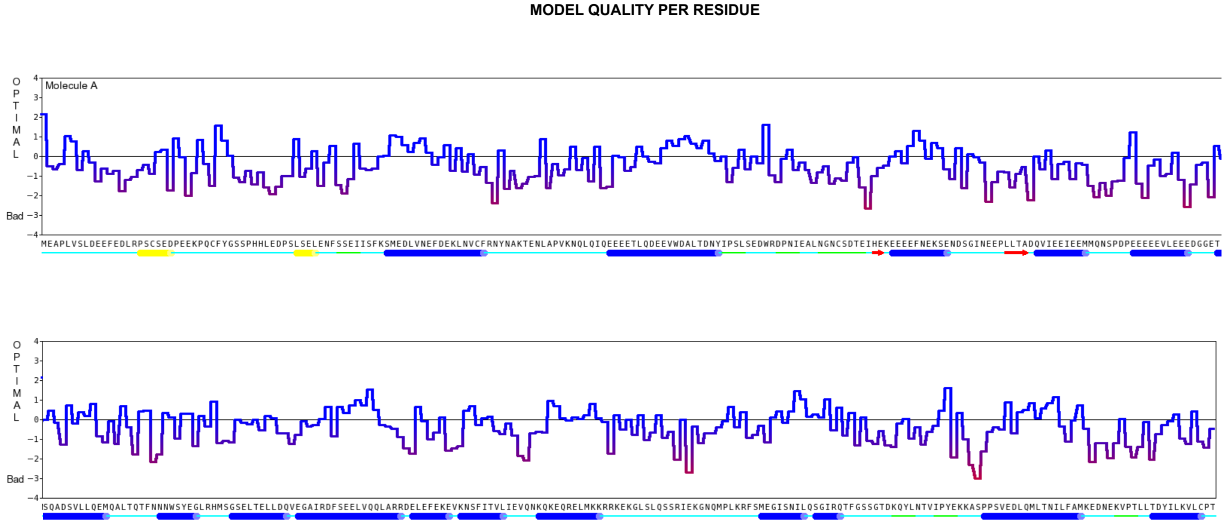

SUPPLEMENTARY FIGURE 5. Per residue Z-score of FEZ1 protein modeling

SUPPLEMENTARY TABLE 1

Supplementary Table 1. Modeled loops in FEZ1 protein

Loop	N-terminal anchor	Loop sequence	C-terminal anchor
1	LQSSR	IEKGNQMPLKRFSMEGISNILQSGIRQTFGSSGTDKQ	YLNTV
2	IHEKE	EEEFNEKSENDSGINE	EPLLT
3	DNEKV	PTLLT	DYILK
4	LQEMQ	ALTQTFN	NNWSY
5	HLEDP	SLSELENFSS	EIISF
6	EIEEM	MQNS	PDPEE
7	ELMKK	RRKEKGLS	LQSSR
8	EVWDA	LTDNYIPSLSEDWRDPNIEALNGN	CSDTE
9	LFAMK	EDN	EKVPT
10	NVCFR	NYNAKTENLAPVKNQLQIQ	EEEET
11	ELEFE	KEV	KNSFI
12	ITVLI	EVQN	KQKEQ
13	WSYEG	LRHMS	GSELT
14	QYLNT	VIPYEKKA	SPPSV
15	DEEFE	DLRPSCSEDPEEKPQCFYGSSPH	HLEDP
16	None	MEAP	LVSLD
